# Factors affecting fatal road crash: the case of insurance, Iran 

**Published:** 2019-08

**Authors:** Ameneh Asadi, Farid Najafi, Mehdi Moradinazar

**Affiliations:** ^*a*^Research Center for Environmental Determinacies of Health, School of Health, Kermanshah University of Medical Sciences, Kermanshah, Iran.

## Abstract

**Background:**

The mortality rate per 10,000 vehicles caused by road traffic accidents is 3 people in the world and 33 people in Iran. This trend has been on the rise in the recent years. Considering the high of mortality rate caused by road traffic accidents in Iran, the present study was carried out to identify the factors influencing the mortality caused by road traffic accidents.

**Methods:**

To analyze the mortality rate caused by road traffic accidents and the factors involved, all closed files in Iran insurance company (compensation unit) in Kermanshah province, Iran in 2013 were investigated. Data analysis was performed by multivariate logistic regression.

**Results:**

From 1517 rood traffic accidents, 2026 people had car crash; 1.33 accidents per each car crash, from whom 1859 (54.1%) people had been injured and 158 (4.6%) had dead. The severity of accident was higher in men than in women, as 102 (68%) men and 48 (32%) women had died due to accident. The most frequent cause of death was brain injuries (67%). Also, 61.4% of the people had died at the site of accident and 29.7% had died during transfer to hospital.

**Conclusions:**

Brain injuries and complications associated with them are the most common causes of death during traffic accidents. More than half of the deaths due to traffic accidents are caused by brain injury. Therefore, protecting the head while driving is one of the most important priorities in traffic accidents. Key words: Traffic Accidents, accident severity, mortality, crash.

**Keywords:**

Traffic Accidents, Accident severity, Mortality, Crash

